# Care after premenopausal risk-reducing salpingo-oophorectomy in high-risk women: Scoping review and international consensus recommendations

**DOI:** 10.1111/1471-0528.17511

**Published:** 2023-05-02

**Authors:** Denise R. Nebgen, Susan M. Domchek, Joanne Kotsopoulos, Joanne A. de Hullu, Emma J. Crosbie, Vincent Singh Paramanandam, Monique M.A. Brood van Zanten, Barbara M. Norquist, Theresa Guise, Serge Rozenberg, Allison W. Kurian, Holly J. Pederson, Nese Yuksel, Rachel Michaelson-Cohen, Sharon L. Bober, Agnaldo Lopes da Silva Filho, Nora Johansen, F. Guidozzi, D. Gareth Evans, Usha Menon, Sheryl A. Kingsberg, C. Bethan Powell, Giovanni Grandi, Claudia Marchetti, Michelle Jacobson, Donal J. Brennan, Martha Hickey

**Affiliations:** 1 Department of Gynecologic Oncology and Reproductive Medicine, University of Texas MD Anderson Cancer Center, Houston, Texas, USA; 2 Basser Center for BRCA, University of Pennsylvania, Philadelphia, Pennsylvania, USA; 3 Women’s College Research Institute, Women’s College Hospital, University of Toronto, Toronto, Ontario, Canada; 4 Department of Obstetrics and Gynaecology, Radboud University Medical Centre, Nijmegen, The Netherlands; 5 Division of Cancer Sciences, University of Manchester, St Mary’s Hospital, Manchester, UK; 6 Department of Obstetrics and Gynaecology, The Royal Women’s Hospital, The University of Melbourne, Melbourne, Victoria, Australia; 7 Department of Gynecology, The Netherlands Cancer Institute, Amsterdam University Medical Centres, Amsterdam, The Netherlands; 8 Department of Obstetrics and Gynecology, University of Washington, Seattle, Washington, USA; 9 Department of Endocrine Neoplasia and Hormone Disorders, University of Texas MD Anderson Cancer Center, Houston, Texas, USA; 10 Department of Obstetrics and Gynaecology, Universite Libre de Bruxelles, Brussels, Belgium; 11 Stanford Cancer Institute, Stanford University School of Medicine, Stanford, California, USA; 12 Case Comprehensive Cancer Center/ University Hospitals Seidman Cancer Center, Cleveland Clinic Taussig Cancer Institute, Cleveland, Ohio, USA; 13 Faculty of Pharmacy and Pharmaceutical Sciences, College of Health Sciences, University of Alberta, Edmonton, Alberta, Canada; 14 Department of Gynaecology and Medical Genetics Institute, Hebrew University Faculty of Medicine, Shaare Zedek Medical Centre, Jerusalem, Israel; 15 Dana-Farber Cancer Institute and Harvard Medical School, Boston, Massachusetts, USA; 16 Departamento de Ginecologia e Obstetricia da UFMG, Federal University of Minas Gerais, Belo Horizonte, Brazil; 17 Department of Gynaecology and Obstetrics, Sørlandet Hospital HF Arendal, Arendal, Norway; 18 Deparment of Obstetrics and Gynaecology, University of Witwatersrand, Johanesburg, South Africa; 19 University of Manchester, Prevent Breast Cancer Centre, Manchester, UK; 20 MRC Clinical Trials Unit, Institute of Clinical Trials and Methodology, University College London, London, UK; 21 University Hospitals Cleveland Medical Center, Case Western University School of Medicine, Cleveland, Ohio, USA; 22 Kaiser Permanente Northern California, Hereditary Cancer Program, San Francisco, California, USA; 23 Department of Medical and Surgical Sciences for Mother, Child and Adult, University of Modena and Reggio Emilia, Modena, Italy; 24 Department of Women’s and Children’s Health, Fondazione Policlinico Universitario A. Gemelli, IRCCS–Catholic University Sacred Heart, Rome, Italy; 25 Women’s College Hospital and Mount Sinai Hospital, University of Toronto, Toronto, Ontario, Canada; 26 UCD Gynaecological Oncology Group, UCD School of Medicine, Mater University Hospital, Dublin, Ireland; 27 Department of Obstetrics and Gynaecology, Research Precinct, Level 7, The Royal Women’s Hospital, University of Melbourne, Parkville, Victoria, Australia

**Keywords:** BRCA1, BRCA2, early menopause, hormone replacement therapy, hot flushes, ovarian cancer, risk-reducing salpingo-oophorectomy, sexual function, surgical menopause

## Abstract

Women at high inherited risk of ovarian cancer are offered risk-reducing salpingo-oophorectomy (RRSO) from age 35 to 45 years. Although potentially life-saving, RRSO may induce symptoms that negatively affect quality of life and impair long-term health. Clinical care following RRSO is often suboptimal. This scoping review describes how RRSO affects short- and long-term health and provides evidence-based international consensus recommendations for care from preoperative counselling to long-term disease prevention. This includes the efficacy and safety of hormonal and non-hormonal treatments for vasomotor symptoms, sleep disturbance and sexual dysfunction and effective approaches to prevent bone and cardiovascular disease.

## 1 Introduction

Carriers of germline pathogenic variants (PVs) in *BRCA1* and *BRCA2* have an approximately 44% and 17% lifetime risk of ovarian cancer or fallopian tube cancer (referred to as ovarian cancer hereafter) and a 72% and 69% lifetime risk of breast cancer.^1^ Surgical removal of both ovaries and fallopian tubes (risk-reducing salpingo-oophorectomy [RRSO]), reduces the risk of ovarian cancer by 80–95% and reduces all-cause mortality in *BRCA1/2* PV carriers.^2,3^ The National Comprehensive Cancer Network (NCCN) recommends that women with *BRCA1* PVs undergo RRSO between 35 and 40 years of age, those with *BRCA2* PVs between 40 and 45 years, and those with *BRIP1, RAD51C* and *RAD51D* between 45 and 50 years.^4^ NCCN recommends that timing of hysterectomy ± RRSO for Lynch syndrome be individualised, as risk varies by PV.^5^ However, patient concerns about the effects of surgical menopause after RRSO are a barrier to risk-reducing oophorectomy.^6^

Prospective studies in premenopausal women undergoing RRSO consistently report an increase in menopausal symptoms including vasomotor symptoms (hot flushes, night sweats), vaginal dryness, sexual dysfunction, and sleep and mood disturbances, which may be persistent and impair quality of life.^7–12^ Prospective studies show that bone density is reduced 2 years after RRSO, which is only partially mitigated by hormone replacement therapy (HRT).^13^ Similarly, HRT decreases but does not fully resolve vasomotor symptoms or sexual dysfunction after RRSO.^8^ In addition, those with a personal history of breast cancer are advised against taking HRT.^14,15^ Effective non-hormonal options are available, but may not be routinely offered. The purpose of this scoping review is to provide clinical recommendations for menopausal symptom management and the prevention of long-term adverse outcomes following RRSO (Table 1). These recommendations were voted on using a modified Delphi questionnaire by a panel of 27 international experts in 12 countries across eight disciplines to form the consensus.

## 2 Methods

**Figure 1:**
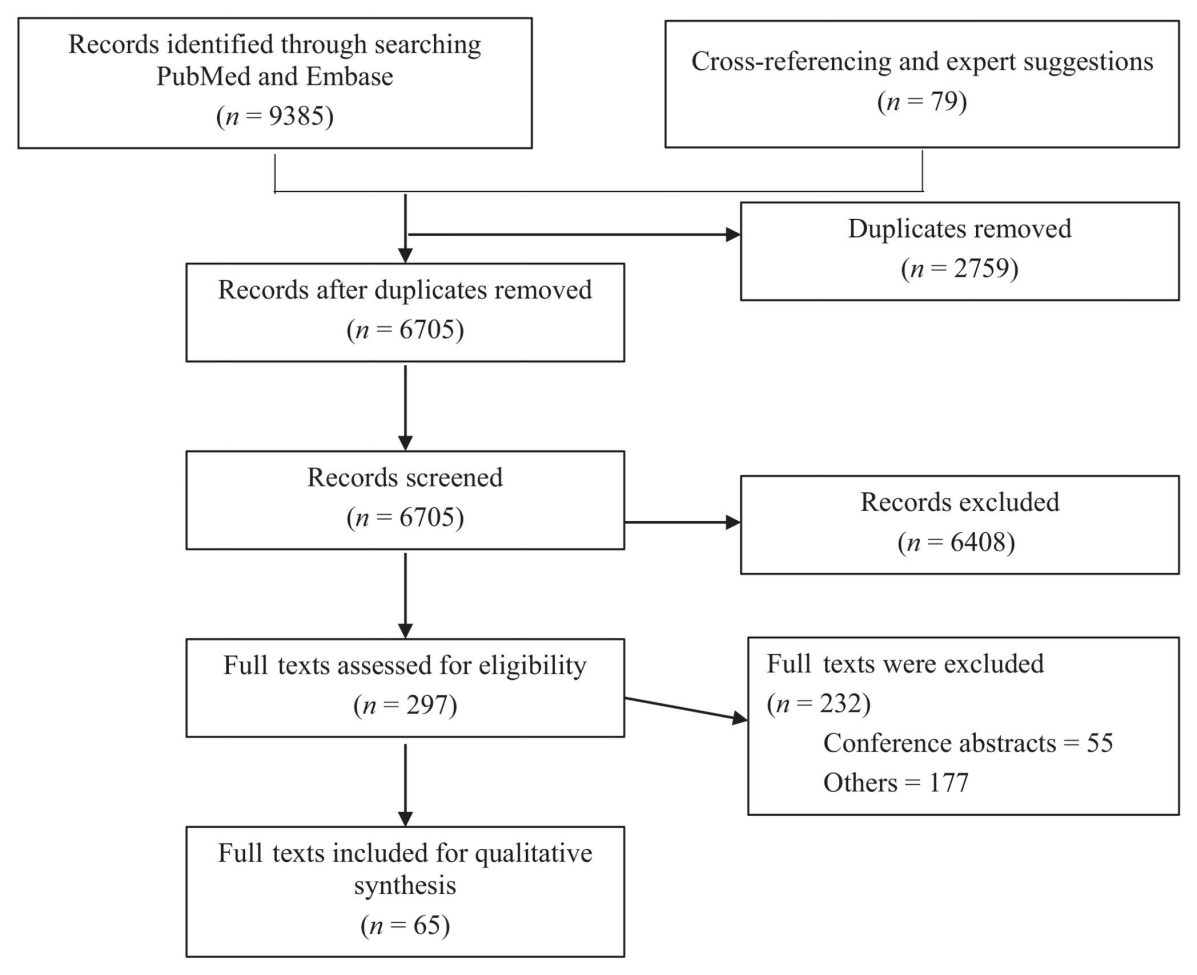
Articles identified and screened for final eligibility.

We searched PubMed database for English-language studies published from inception to January 2021 using a scoping review process (search terms are reported in eAppendix in Data S1). Two reviewers (MH, DRN) independently screened 6705 publications first by titles and abstracts, and then 297 full-text articles from which 65 publications were included (Figure 1). Characteristics of these 65 including design and country of origin are summarised in Table 2. Most were from North America (35; 46%), followed by multicontinental collaborations (24; 32%) and Europe (10; 13%). Most were cohort studies (21; 28%), followed by randomised controlled trials (RCTs) (13; 17%) and systematic reviews (11; 15%).

### 2.1 Scoping review

Scoping reviews are recommended for interrogating and summarising the literature when there is more than one research question. The main difference between a scoping review and a systematic review is that systematic reviews address one particular question, whereas a scoping review addresses several questions from a large and diverse body of literature pertaining to a broad topic. Hence, a scoping review (rather than a systematic review) was the most rigorous approach to evaluate the evidence and inform these clinical recommendations. This scoping review included 6705 published papers, which formed the basis of these consensus recommendations. We have followed the preferred framework for a scoping review: (1) identifying the research question, (2) identifying relevant studies, (3) study selection, (4) charting the data, (5) collating, summarising and reporting the results, and (6) an optional consultation exercise.^16^ As recommended, the clinical recommendations have been graded based on the level of evidence they were derived from.

### 2.2 Consensus process

Two authors (MH and DRN) contacted the first and/or senior authors from 65 publications identified in the scoping review that reported short- or long-term health outcomes following RRSO or care of high-risk women following RRSO to invite them to contribute to the consensus process. Of these, 27/29 responded and agreed to participate. The authors represent eight disciplines involved in the care of PV carriers such as *BRCA1/BRCA2* from 12 countries. Consensus was achieved using an online modified Delphi Survey including 50 proposed recommendations based on data synthesised from the scoping review, with grading of the evidence using traditional Ia–IV ratings, shown in Table 1. For each question they were asked to rate their responses on a Likert scale from 1 to 9. Statements were Strongly Recommend (SR) when more than 80% of participants scored as ‘7–9’ and less than 10% scored as ‘1–3’, Recommended (R) when more than 70% scored as ‘7–9’ and less than 15% scored as ‘1–3’, and Not Recommended (NR) when more than 70% scored as ‘1–3’ and less than 15% scored ‘7–9’. Neutral statements (N) did not meet any of these criteria. Twenty-two consensus statements were scored as (SR), 8 were (R) and 21 were (N). None were classed as (NR). Clinically relevant consensus recommendations are listed in Table 1. Most of the consensus process occurred during the pandemic and formal meetings between the authors were not possible. Communications were primarily by email and online surveys. Virtual meetings were held as needed.

### 2.3 Search strategy and selection criteria

We searched PubMed for publications from inception to January 2021, using the search terms ‘RRSO’, ‘risk reducing bilateral salpingo-oophorectomy’, ‘risk reducing salpingectomy’, ‘breast cancer gen’e’, ‘BRCA’, ‘cardiovascular disease’, ‘mood’, ‘sexual dysfunction’, ‘sleep’, ‘vasomotor symptoms’, ‘bone density’, ‘osteopenia’, ‘osteoporosis’, ‘premature ovarian insufficiency’, ‘early menopause’, ‘surgical menopause’, ‘hormone replacement therapy’, ‘menopausal hormone therapy’, ‘genitourinary’ and ‘non-cancer endpoints’ restricted to articles in English. Duplicate articles from the database search were removed using Endnote (Endnote 18, Clarivate™), and uploaded to Covidence (Covidence) for screening (Appendix in Data S1).

## 3 Preoperative Counselling of High-Risk Women Before RRSO

Common concerns for women facing RRSO include loss of fertility, management of menopausal symptoms, impact on sexual function and long-term health.^6^ It was strongly recommended by the panel that these issues be discussed before RRSO to ensure informed consent, set realistic expectations and generate an individualised plan for post-operative care. High-risk women making decisions about surgery to reduce their risk of ovarian cancer may benefit from a Decision Aid, and patient information resources addressing the consequences and management of surgical menopause after RRSO are available to download.^17,18^ Referral to a fertility specialist should be offered where relevant.

- Offer women who have not completed childbearing referral to a fertility specialist—SR
- Address patient concerns about loss of fertility, menopausal symptoms, sexual function and long-term health^6^—SR

### 3.1 Discussion of concurrent hysterectomy at RRSO

Hysterectomy is advised for women with Lynch syndrome where endometrial cancer risk is significantly elevated and the efficacy of screening is uncertain.^5,19^ Decision-making about hysterectomy at the time of RRSO for *BRCA1* and *BRCA2* is more complex. Two systematic reviews and meta-analyses on endometrial cancer risk in *BRCA1* and *BRCA2* carriers show a slight increased relative risk of serous endometrial cancer in *BRCA1* (Standardised Incidence Ratio 2.81) and *BRCA2* (Standardised Incidence Ratio 1.75)^20,21^ but minimal increase in absolute risk. However, a recent (2022) study of 5341 families with PVs in *BRCA1* and *BRCA2* shows no increased risk of endometrial cancer.^22^ In light of these findings, NCCN guidelines advise discussion of the benefits and risk of hysterectomy but do not recommend routine hysterectomy in *BRCA* PV carriers.

## 4 Counselling and Care of High-Risk Women After RRSO

The overall aim after RRSO is prevention of adverse health consequences from early surgical menopause and optimisation of long-term physical and emotional health.^14^

## 5 Management of Vasomotor Symptoms Following RRSO in Women Without Breast Cancer

Around 80% of premenopausal women develop vasomotor symptoms after RRSO.^8^ Cross-sectional studies report more severe vasomotor symptoms after RRSO compared with natural menopause, which may be persistent.^23^ HRT may be less effective for vasomotor symptoms after RRSO. In the general population, HRT reduces vasomotor symptoms by around 80%, but following RRSO around 40% report persistent symptoms despite HRT use.^8^ Uptake of HRT after RRSO varies widely across the country ranging from 5% to 75%, but on average 50–60% of women take HRT after RRSO.^24^ The long-term safety of HRT for breast cancer in this population is not known. A prospective study of 872 *BRCA1* PV carriers of whom 43% (77% premenopausal, 23% postmenopausal at RRSO, mean age 43 years, range 30–70 years) took HRT for a mean of 3.9 years (range 0.5–19 years) concluded that estrogen alone did not increase breast cancer risk but the safety of progestin-containing HRT was uncertain.^25^ A 2019 systematic review including seven studies (*n* = 933 *BRCA1/2* PV carriers) concluded that HRT could be used for up to 4.3 years without increasing breast cancer risk.^26^ Age at initiation of HRT may be important. A retrospective study of 306 *BRCA1/2* PV carriers followed up for up to 7.26 years after RRSO found no overall increased risk of breast cancer after 4 years of HRT; however, those who started HRT after age 45 years had a more than three-fold increased risk of breast cancer (odds ratio 3.43, *p* < 0.05, 95% CI 1.2–9.8).^27^ Whether this reflects higher risk of BC with age or is a direct effect of HRT is unknown. This information should be balanced with the potential cardioprotective effects of HRT if taken until the age of 50 years, after early oophorectomy.^28^ Together, these data support offering HRT to women under age 45 years with duration of use dependent on previous mastectomy and/or hysterectomy, patient wishes, severity of menopausal symptoms and risk factors for osteoporosis. However, more information is needed about morbidity and mortality associated with HRT use in high-risk women and little is known about safety beyond 4–5 years of use.

If progestin is needed, limited observational data in the general population suggest that micronised progesterone might have a lower risk of breast cancer compared with synthetic progestins, but there are no studies in *BRCA1/2* PV carriers.^29^ Intrauterine progestin via the levonorgestrel intrauterine system has minimal systemic absorption but is still associated with elevated breast cancer risk (relative risk 1.19, 95% CI 1.13–1.25) in the general population.^30^ Data from the general population suggest that those with risk factors for venous thromboembolism should be offered transdermal rather than oral estrogen.^31^

- Offer HRT following RRSO below age 45 years for vasomotor symptoms and/or disease prevention^25,26^—SR
- After age 45 years, individualise HRT considering need for progestin, previous risk reducing mastectomy, severity of symptoms, and risk factors for osteoporosis—SR
- Offer transdermal HRT in those at elevated risk of venous thromboembolism^31^—R
- Consider micronised progesterone instead of synthetic progestogen^29^—N

## 6 Management of Vasomotor Symptoms Following RRSO in Women with Breast Cancer

A 2021 systematic review and meta-analysis in the general population (*n* = 4050) showed a two-to three-fold increased risk of recurrent or new breast cancer in HRT users after estrogen receptor-positive but not estrogen receptor-negative breast cancer.^32^ When HRT is contraindicated or avoided there are several effective non-pharmacological and non-hormonal treatments for vasomotor symptoms.^33,34^

### 6.1 Non-pharmacological treatments for vasomotor symptoms (Figure 2)

Randomised controlled trials show that cognitive behavioural therapy (CBT) reduces the bother/impact of vasomotor symptoms in the general population and after breast cancer.^35^ Following RRSO, mindfulness-based stress reduction improves quality of life for patients with vasomotor symptoms.^36^ One RCT in the general population showed that clinical hypnosis reduced vasomotor symptoms.^33^ Some RCTs show that acupuncture reduces vasomotor symptoms after breast cancer, but data are mixed.^33^

- Offer CBT for vasomotor symptoms^35,37^—N
- Consider mindfulness-based stress reduction^36^—N

### 6.2 Non-hormonal pharmacological treatments for vasomotor symptoms (Figure 2)

Evidence from RCTs in breast cancer patients demonstrates that effective non-hormonal pharmacological therapies for vasomotor symptoms include selective serotonin reuptake inhibitors (SSRI) and serotonin–norepinephrine reuptake inhibitors (SNRI), gabapentin, clonidine and oxybutynin, although studies are lacking after RRSO.^33,34^ No single agent has proven superior to others, but SSRI and SNRI reduce vasomotor symptoms by around 50–60%. A systematic review of non-hormonal treatments for vasomotor symptoms after breast cancer showed similar outcomes for 10/20/30 mg citalopram and 10/20 mg paroxetine, venlafaxine 75 mg daily and gabapentin 900 mg.^38^ Escitalopram 10/20 mg is also effective.^39^ Clonidine reduces vasomotor symptoms by about 40%.^33,34^ Oxybutynin 2.5–5 mg twice a day is also effective, although adverse effects may limit its use.^33^ There is a hypothetical risk that fluoxetine and paroxetine may interfere with tamoxifen metabolism but this does not appear to impact on breast cancer outcomes.^40^

- Offer SSRI (paroxetine 10/20 mg; citalopram 10/20/30 mg; escitalopram 10/20 mg) or SNRI (venlafaxine 75 mg controlled release or desvenlafaxine 100 mg)^38,39^—N
- Offer gabapentin 300–900 mg at night for vasomotor symptoms that disturb sleep^38^—N
- Consider clonidine 0.1-mg patch^33,34^—N
- Consider oxybutynin 2.5–5 mg twice a day^33^—N
- Consider hypnosis or acupuncture^33^—N

## 7 Sleep Distur Bance After RRSO

Sleep disturbance is common over the menopause transition and may be independent of vasomotor symptoms. A retrospective study in the general population suggested that sleep quality was worse after surgical menopause compared with natural menopause.^41^ In a prospective study, following RRSO, HRT improved sleep quality but not to baseline levels.^9^ Sleep disturbance may increase the risk of mood disturbance at menopause and requires active management. High-level evidence in the general population supports the efficacy of CBT for insomnia (CBT-I).^42^ Care should include advice about sleep hygiene including avoidance of caffeine and alcohol, reducing noise, routine exercise and maintaining a regular sleep schedule.^43^ Following breast cancer, both gabapentin and acupuncture improve sleep.^44^ The addition of zolpidem 5–10 mg to SSRI improves sleep and quality of life after breast cancer.^45^ One RCT in breast cancer survivors showed that melatonin 3 mg improves subjective sleep quality without significant adverse effects.^46^

- Advise about good sleep hygiene including avoidance of caffeine and alcohol, eliminate noise from bedroom, get routine exercise and maintain a regular sleep schedule^43^—R
- Offer CBT Insomnia programmes available on-line^42^—N
- Offer HRT to eligible women with sleep disturbance due to vasomotor symptoms^9^—SR
- Consider gabapentin 300–900 mg at night to improve sleep quality and duration^44^—N
- Consider augmentation of SSRI and SNRI with zolpidem 5–10 mg^45^—N
- Consider melatonin 3 mg^46^—N

## 8 Mood Disturbance After RRSO

Women with previous major depressive disorder are at risk of relapse over the menopause transition.^47,48^ Other risk factors for depression include anxiety, stressful life events, vasomotor symptoms and poor sleep.^48^ HRT does not prevent or treat depression over the menopause transition. However, CBT as shown in general population women, reduces depressive symptoms and improves sleep.^37^ Consensus guidelines for the general population indicate that mood disturbances should be managed with standard approaches such as antidepressants and/or psychotherapy and refer if indicated.^48^

- Be aware that previous depressive illness increases risk of recurrent depression^47^—SR
- Consider CBT for women with vasomotor and depressive symptoms^37^—R
- Consider SSRI/SNRI for vasomotor symptoms and depressive symptoms—N
- Refer for major depressive disorder or anxiety disorder—SR

## 9 Sexual Dysfunction After RRSO

Several cross-sectional studies report a high prevalence of sexual dysfunction after RRSO, which may be worse after breast cancer or treatment with endocrine therapy.^7,49^ Sexual problems after RRSO include vaginal dryness and dyspareunia, reduced libido, reduced arousal and difficulty with orgasm. Preoperative counselling about sexual function may reduce subsequent distress after RRSO.^50^ Risk factors for sexual dysfunction in *BRCA* PV carriers after RRSO include depression, vasomotor symptoms, poor sleep and sexual inactivity.^51^ HRT improves but does not resolve sexual dysfunction after RRSO.^11,12^ SSRI and SNRI may cause sexual adverse effects, including difficulty reaching orgasm or hypoactive sexual desire disorder (HSDD). In the general population, a 2014 systematic review reported that vaginal estrogens effectively relieve common genito-urinary symptoms associated with menopause.^52^ A systematic review in 2020 of vaginal estrogen safety reported that these products appear safe for at least 1 year of use.^53^ Although vaginal estrogens are systemically absorbed, circulating concentrations remain in the postmenopausal range.^53^ In the general population, long-term follow-up studies do not show any increase in breast or endometrial cancer with vaginal estrogen use.^54^ Although the safety and efficacy of vaginal estrogen in *BRCA1/2* pathogenic variant carriers has not been studied, high rates of sexual dysfunction in this population suggest that vaginal estrogens should be considered for troublesome genitourinary symptoms such as vaginal dryness in those without a personal history of breast cancer.^49^ After estrogen-dependent breast cancer, American College of Obstetricians and Gynecologists guidelines advise using non-hormonal methods as first line for genitourinary symptoms, but to consider vaginal estrogen if these are ineffective, following discussing with the treating oncologist.^55^

In the general population, vaginal dehydroepiandrosterone (DHEA; prasterone) improves vaginal atrophy, sexual satisfaction and dyspareunia without increasing circulating estradiol or testosterone.^56^ The North American Menopause Society Global position statement advises assessment for HSDD using the Decreased Sexual Desire Screener.^57^ Topical testosterone is recommended for HSDD in the general population, but no studies have evaluated its safety in *BRCA1/2* carriers.^58,59^

Flibanserin and bremelanotide are US Food and Drug Administration (FDA) approved non-hormonal therapies for HSDD in premenopausal women and have similar efficacy and use in postmenopausal women, and can be considered.^60,61^ In one study after RRSO, an education session including sexual health, relaxation training, body awareness and CBT improved overall sexual functioning.^62^

- Before RRSO, discuss the potential detrimental effects on sexual function which may be long-lasting and not restored by HRT^11,12^—SR
- Ask about sexual activity and satisfaction after RRSO—SR
- Review risk factors for sexual dysfunction such as depression, vasomotor symptoms, poor sleep and sexual inactivity^51^—SR
- If HSDD, consider topical testosterone therapy^58^—N
- Since in many countries female testosterone products do not exist, use one-tenth of a male FDA-approved product (best in tube form)^59^—N
- Do not use testosterone compounded or pellets^58^—R
- Consider vaginal prasterone (DHEA) for vaginal atrophy and dyspareunia^56^—N

## 10 Genitourinary Symptoms After RRSO

Around 50% of postmenopausal women report genitourinary symptoms, which may include dryness, dyspareunia, urinary urgency/frequency, and vulvar and vaginal burning. A systematic review of 53 RCTs in the general population showed that vaginal estrogen improves vaginal dryness and may also prevent recurrent urinary tract infection.^63,64^ Effective non-hormonal therapies include hyaluronic acid containing moisturisers and lubricants but these may not be as effective as estrogen.^65^ After breast cancer, US guidelines advise that vaginal estrogen be considered if non-hormonal therapies are ineffective, in consultation with the medical oncologist.^55^ Vaginal prasterone and oral ospemifene are also effective but not licenced after breast cancer.^56^ A sham-controlled RCT of vaginal CO_2_ laser in the general population showed no benefit for any genitourinary symptoms including dyspareunia.^66^ Another RCT reported that 4% aqueous lidocaine before penetrative intercourse allowed 17/20 (85%) breast cancer survivors who previously abstained from intercourse to resume sexual activity.^67^

- Consider non-hormonal treatments including hyaluronic acid containing vaginal moisturisers and lubricants^33,65^—SR
- Offer vaginal estrogen for vaginal dryness^63^—SR
- Discuss vaginal estrogen with oncologist if previous breast cancer^55^—R
- Consider topical 4% lidocaine for dyspareunia in breast cancer survivors^67^—N
- Do not offer vaginal laser^66^—N

## 11 Cardiovascular Health After RRSO

Cardiovascular disease (CVD) is the leading cause of death in men and women worldwide. *BRCA1/2* PV carriers may be at higher risk of CVD as the result of early menopause, chest radiotherapy or chemotherapy for breast cancer.^68^ Surgical menopause may further increase CVD risk. Pooled data from 203 767 women in the general population reported a higher risk of CVD following surgical menopause compared with women of similar age with spontaneous early menopause, with greater risk in younger age at oophorectomy.^69^ Use of HRT until age 50 years reduced CVD risk.^69^ In the Nurse’s Health Study, a prospective cohort study of 30 000 women undergoing hysterectomy for benign disease, bilateral oophorectomy was associated with increased mortality in women aged younger than 50 years who did not take estrogen therapy.^28^ A cross-sectional study of 165 *BRCA1/2* PV carriers, 5–24 years after RRSO (at <45 years) showed no evidence of increased CVD.^11^ Sedentary behaviour and physical inactivity are the leading modifiable risk factors for CVD worldwide.^70,71^ Other modifiable risk factors include obesity, smoking, hypertension, elevated lipids and diabetes. The cardioprotective effects of regular physical activity in the general population are clear and extend across all ages, sex and race.^71^ Evidence-based guidelines from the general population to optimise CV health should be applied to the *BRCA1/2* population. According to the Physical Activity Guidelines of the US Department of Health and Human Services, adults should reduce sedentary activity and aim for moderate aerobic exercise of 150–300 minutes/week, or vigorous aerobic exercise of 75–150 minutes/week and strength training 2 days per week to improve overall health and reduce risk of CVD.^70^

- Consider HRT for disease prevention in those under age 50 years at RRSO^28,69^—SR
- Check annual weight and blood pressure—SR
- Minimise sedentary behaviour, improve diet, decrease alcohol, stop smoking^70^—SR
- Aim for 150–300 minutes/week of moderate aerobic exercise or 75–150 minutes/week of vigorous aerobic exercise and strength training (2×/week)^70^—SR

## 12 Bone Health After RRSO

In the general population, premature ovarian insufficiency reduces bone mineral density and may increase fracture risk.^72^ Use of HRT may prevent or minimise bone loss.^72^ A retrospective chart review (*n* = 225) following RRSO demonstrated that 56% had osteopenia and 12% had osteoporosis.^73^ Another retrospective cohort study after RRSO suggested that HRT users had higher bone density than non-users.^74^ Prospective studies after RRSO suggest that HRT improves bone density and strength but not to baseline levels.^13,75^ The optimal dose and delivery system of HRT to prevent osteoporosis and fracture is not known.

The 2020 update of the American Association of Clinical Endocrinologists clinical practice guidelines on osteoporosis considers early menopause to be an indication for baseline bone mineral density with dual-energy X-ray absorptiometry (DXA) testing within the first year with repeat 1–2 years later.^76^ Endocrinology referral should be considered for significant osteopenia, osteoporosis or for women at risk of minimal trauma fracture.^76^ If baseline DXA shows mild osteopenia, repeat every 2 years thereafter or if normal, a repeat study should be considered in 3–5 years or based on healthcare system reimbursement. If bone mineral density remains normal until age 50 years, consider stopping DXA and resume at age 65 years according to general population guidelines. The Fracture Risk Assessment Tool (FRAX) can be used to calculate 10-year fracture risk.^76^ Modifiable risk factors for osteoporosis include low body mass index (<18 kg/m^2^), low physical activity, insufficient calcium and vitamin D intake, insufficient protein intake, smoking, and excessive consumption of alcohol (>2 drinks/day). Management to detect and prevent osteoporosis include monitoring bone mineral density and aiming for normal vitamin D levels (>30 ng/mL; 75 nmol/L) with adequate vitamin D intake (600–800 IU/day)^76,77^ and adequate calcium intake (1000–1200 mg/day) along with limiting caffeine and alcohol and smoking cessation. Bone strengthening and fall prevention include maintaining an active lifestyle including routine aerobic, weight-bearing (3–5×/week), balance and muscle-strengthening (2–3×/week) exercises.^76,77^

- Offer HRT to those without contraindications^13^—SR
- Advise routine aerobic, weight bearing (3–5×/week), balance and strength training (2–3×/week) exercises (weight bearing is walking, jogging, jumping rope or on your feet equivalent)^76,77^—SR
- Counsel on adequate dietary or supplementary calcium (1000–1200 mg/day) and vitamin D (600–800 IU/day) or according to national guidelines^76^—SR
- Order DXA following premenopausal RRSO within first year^76^—SR
- If initial DXA shows significant osteopenia or osteoporosis, refer to bone health specialist^76^—R
- If initial DXA shows osteopenia repeat every 2 years or if normal, consider repeat in 3–5 years or based on healthcare system reimbursement^76^—R
- If at high risk of minimal trauma fracture, refer to bone health specialist^76,77^—SR
- Aim for normal vitamin D levels (>30 ng/mL; 75 nmol/L)^77^—N
- If vitamin D is low, offer replacement—N
- Calculate FRAX in women over 40 years^77^—R
- If back pain or height loss greater than 2 cm, advise lateral thoracic/lumbar spine X-ray^77^—N
- Consider bone-protective therapy and refer to bone specialist if:^76,77^—SR
  - Previous hip, fragility or clinical vertebral fracture
  - DXA femoral neck, total hip or spine *T* score is less than −2.5 (osteoporosis)
  - Osteopenia+10-year hip fracture risk of more than 3% on FRAX
  - Osteopenia+10-year major osteoporosis-related fracture risk of more than 20% on FRAX

## 13 Cognitive Function and RRSO

It is not known whether surgical menopause affects cognition. A systematic review of 11 studies (*n* = 18 867 women) in the general population found conflicting results from low-quality studies and concluded that surgical menopause before age 45 years may increase dementia and cognitive decline.^78^ Following RRSO, one small cross-sectional study reported a small subjective but not objective decline in cognition at 6 months that was not prevented by HRT.^79^ More information from prospective studies of cognition after RRSO is needed. Recommendations for the prevention of dementia and effects of HRT are unanswered.

## 14 Uncertainties

There are numerous areas of uncertainty around RRSO in *BRCA1/2* pathogenic variant carriers. After RRSO, the optimal dose, route of administration and duration of HRT use are unknown. Similarly, it is uncertain whether HRT can be safely used by women with a personal history of estrogen receptor-negative breast cancer who have undergone bilateral mastectomy. The views of high-risk women were not formally sought in this consensus process which is a limitation. How best to optimise long-term health and mitigate the risk of chronic disease after RRSO requires long-term prospective studies.

## 15 Conclusions

Despite the efficacy of premenopausal RRSO for reducing ovarian cancer risk and improving mortality in high-risk women, many women experience troublesome menopausal symptoms and oophorectomy may have adverse implications for long-term health. Our panel of international experts has developed evidence-based recommendations for managing vasomotor, sleep, mood, sexual, and genitourinary symptoms and optimising bone and cardiovascular long-term health. Emerging evidence suggests that HRT reduces but does not eliminate the adverse effects of premenopausal oophorectomy. Women and clinicians considering RRSO should be aware of these risks and clinical care should focus on available safe options for symptom management and optimisation of long-term health.

## Supplementary Material

Supplementary Materials

## Figures and Tables

**Figure 2:**
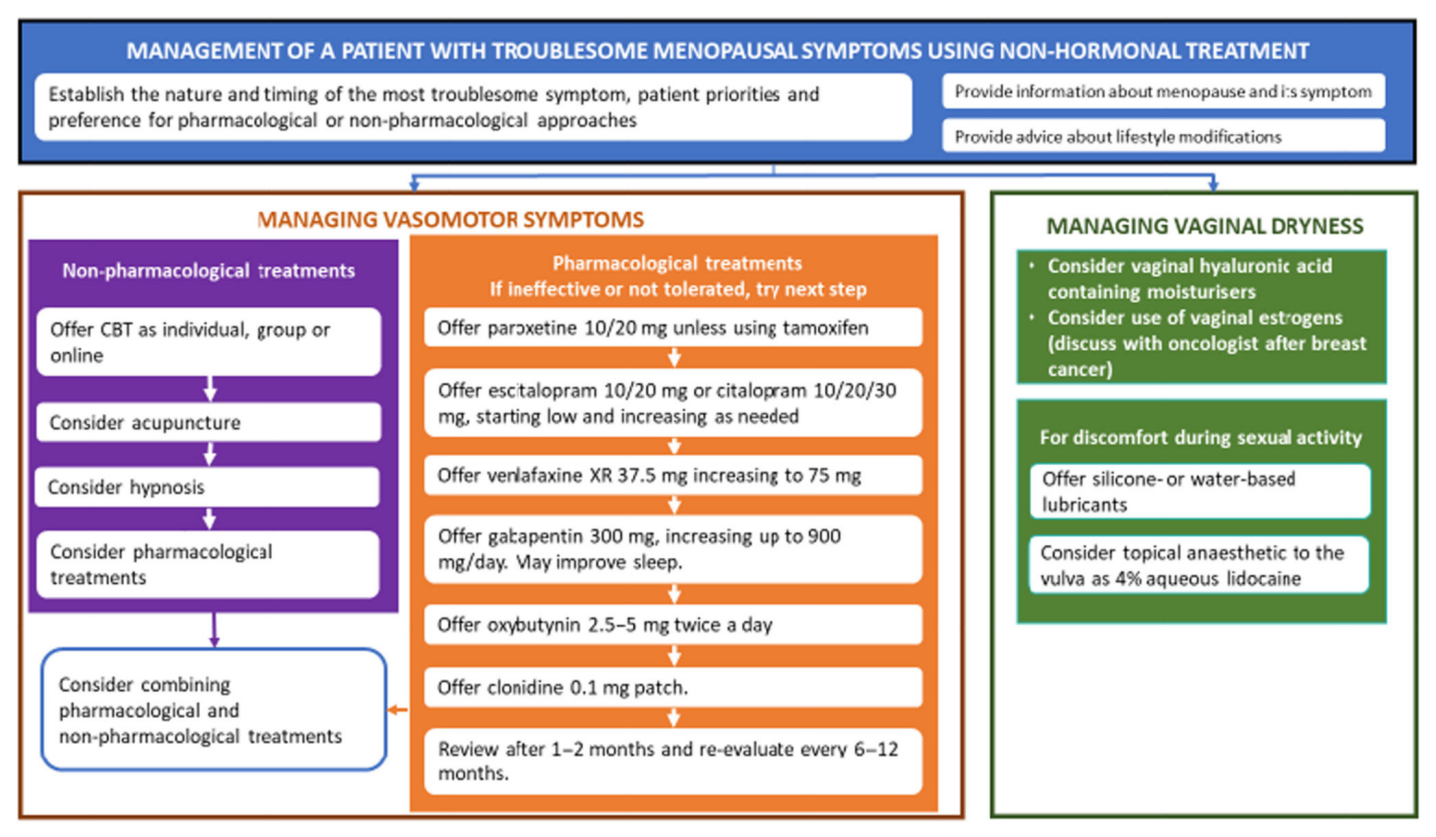
Management of problematic menopausal symptoms using non-hormonal treatment.^30^

**Table 1:** Care after premenopausal risk reducing salpingo-oophorectomy in high risk women: international consensus recommendations.

Level	Description		Rationale
Strongly Recommend – SR	Patients should expect this level of care		Unanimous agreement from the consensus members
Recommend – R	Care providers and stakeholders should aim to provide this level of care		Unanimous agreement from the consensus members
Neutral – N	Care providers and stakeholders may wish to provide this service		No unanimous agreement
	**References**	**Level of evidence**	**Rec**
Discussion before RRSO			
Offer women who have not completed childbearing referral to a fertility specialist		IV (C)	SR
Address patient concerns about loss of fertility, menopausal symptoms, sexual function and long-term health	[6]	III (B)	SR
Management of vasomotor symptoms (VMS) following RRSO in women without breast cancer			
Offer HRT below age 45 years for vasomotor symptoms and/or disease prevention	[25,26]	IIa (B)	SR
After age 45 years, individualise HRT considering need for progestin, previous risk-reducing mastectomy, severity of symptoms and risk factors for osteoporosis		IV (C)	SR
Offer transdermal HRT in those at elevated risk of VTE	[31]	IIa (B)	R
Management of VMS following RRSO in women with breast cancer			
Offer non-hormonal treatments for troublesome VMS—Figure 2			
Management of sleep disturbance after RRSO			
Advise about good sleep hygiene including avoidance of caffeine and alcohol, eliminate noise from bedroom, get routine exercise and maintain a regular sleep schedule	[43]	IIb (B)	R
Offer HRT to eligible women with sleep disturbance due to VMS	[9]	IV (C)	SR
Management of mood disturbance after RRSO			
Be aware that previous depressive illness increases risk of recurrent depression	[47]	IIb (B)	SR
Consider CBT for women with vasomotor and depressive symptoms	[37]	Ib (A)	R
Refer for major depressive disorder or anxiety disorder		IV (C)	SR
Management of genitourinary symptoms and sexual dysfunction after RRSO			
Before RRSO, discuss the potential detrimental effects on sexual function, which may be long-lasting and not restored by HRT	[11,12]	IIa (B)	SR
Ask about sexual activity and satisfaction after RRSO		IV (C)	SR
Review risk factors for sexual dysfunction such as depression, vasomotor symptoms, poor sleep and sexual inactivity	[51]	III (B)	SR
Offer vaginal estrogen for vaginal dryness	[63]	1b (A)	SR
Discuss with oncologist if previous breast cancer	[55]	IV (C)	R
Consider non-hormonal treatments including hyaluronic acid containing vaginal moisturisers and lubricants	[33,65]	IIb (B)	SR
Do not use testosterone compounded or pellets	[58]	IV (C)	R
Management of CVD risk after RRSO			
Consider HRT for prevention in those under age 50 years at RRSO	[28,69]	III (B)	SR
Check annual weight and blood pressure		IV (C)	SR
Minimise sedentary behaviour, improve diet, decrease alcohol and stop smoking	[70]	IIa (B)	SR
Aim for 150–300 min/week of moderate aerobic exercise or 75–150 min/week of vigorous aerobic exercise and strength training (2×/week)	[70]	IIa (B)	SR
Prevention of bone disease after RRSO			
Offer HRT to those without contraindications	[13]	IIa (B)	SR
Advise routine aerobic, weight bearing (3–5×/week), balance, and strength training (2–3×/week) exercises (weight bearing is walking, jogging, jumping rope or on your feet equivalent)	[76,77]	Ib (A)	SR
Counsel on adequate dietary or supplementary calcium (1000–1200 mg/day) and vitamin D (600–800 IU/day) or according to national guidelines	[76]	IIa (B)	SR
Order DXA following premenopausal RRSO within first year	[76]	IIa (B)	SR
If initial DXA shows significant osteopenia or osteoporosis, refer to bone health specialist	[76]	IV (C)	R
If initial DXA osteopenia repeat every 2 years or if normal, consider repeat in 3–5 years or based on healthcare system reimbursement	[76]	IV (C)	R
If at high risk of minimal trauma fracture, refer to bone health specialist	[76,77]	IIb (B)	SR
Calculate FRAX in women over 40 years	[77]	IIa (B)	R
Consider bone-protective therapy and refer to bone specialist if	[76,77]	Ib (A)	SR
Previous hip, fragility or clinical vertebral fracture DXA femoral neck, total hip or spine T score less than ȡ2.5 (osteoporosis) osteopenia+10-year hip fracture risk of more than 3% on FRAX osteopenia+10-year major osteoporosis-related fracture risk of more than 20% on FRAX			
**Category of evidence**	**Grading of evidence**		
	Traditional guideline		
Meta-analysis of randomised controlled trials	Ia A		
Randomised controlled trials	Ib A		
Well-designed and controlled study without randomisation	IIa B		
Well-designed quasi-experimental study	IIb B		
Non-experimental descriptive study	III B		
Expert opinion	IV C		

**Table 2:** Study design and country of origin.

	Counselling (%)	Mx of symptoms without BC (%)	Mx of symptoms with BC (%)	Non-pharmacology (%)	Non-hormonal (%)	Sleep disturbance (%)	Mood disturbance (%)	Sexual dysfunction (%)	GU symptoms (%)	CV health (%)	Bone health (%)	Cognitive function (%)
Place country?												
A (16, 18, 24, 36) AU (45, 59)	1(14)	2(15)				1 (13)		1 (8)	1 (17)			
EU (11, 14, 20, 23, 26, 28, 31, 32, 71)	1 (14)	4 (31)	1 (25)	2 (40)				1 (8)				1 (100)
NA (4, 5, 7, 15, 25, 33– 35, 37–44, 46, 47, 52–56, 58, 60, 63, 66–70, 72)	2 (29)	2 (15)	1 (25)	2 (40)	3 (75)	5 (63)	4 (67)	7 (54)	3 (50)	1 (33)	5 (83)	
SA (27)		1 (8)										
Others* (8–10, 12, 13, 16, 17, 21, 22, 29, 30, 48, 50, 51, 62, 74)	3 (43)	4 (31)	2 (50)	1 (20)	1 (25)	2 (25)	2 (33)	4 (31)	2 (33)	2 (67)	1 (17)	
Study design												
Chart review (66)											1 (17)	
Cohort study (7–10, 12, 13, 21, 22, 24, 26, 35, 36, 42,62, 67, 74)	1 (14)	7 (54)			1 (25)	3 (38)	3 (50)	2 (15)		2 (67)	2 (33)	
Cross-sectional (20, 45, 47)		1 (8)						2 (15)				
Experimental (11, 54, 72)		1 (8)		1 (20)				2 (15)				
Guidelines (4, 5, 15, 43, 48,50, 51, 58, 63, 69, 70, 75)	2 (29)	1 (8)					1 (17)	3 (22)	2 (33)	1 (33)	2 (33)	
Qualitative (46)								1 (8)				
RCT (32, 34, 39–41, 44, 52, 53, 59, 60, 68)			1 (25)	1 (20)	1 (25)	3 (38)	2 (33)	3 (23)	1 (17)		1 (17)	
Review (14, 23, 29–31, 38, 55)	1 (14)	1 (8)	2 (50)	2 (40)	1 (25)	1 (13)			2 (33)			
SR (16–18, 25, 27, 28, 33, 37, 56, 71)	3 (43)	2 (15)	1 (25)	1 (20)	1 (25)	1 (13)			1 (17)			1 (100)

Abbreviations: A, Asia; AU, Australasia; BC, breast cancer; CV, cardiovascular health; EU, Europe; GU, genitourinary; Mx, management; NA, North America; RCT, randomised controlled trial; SA, South America; SR, systematic reviews, (%)

* Multinational collaboration.

## Data Availability

Data openly available in a public repository that issues datasets with DOIs. The data that support the findings of this study are openly available in [https://pubmed.ncbi.nlm.nih.gov/].
